# Methylene Blue Protects Astrocytes against Glucose Oxygen Deprivation by Improving Cellular Respiration

**DOI:** 10.1371/journal.pone.0123096

**Published:** 2015-04-07

**Authors:** Gourav Roy Choudhury, Ali Winters, Ryan M. Rich, Myoung-Gwi Ryou, Zygmunt Gryczynski, Fang Yuan, Shao-Hua Yang, Ran Liu

**Affiliations:** 1 Department of Pharmacology and Neuroscience, Institute for Aging and Alzheimer’s Disease Research, University of North Texas Health Science Center, Fort Worth, Texas, United States of America; 2 Department of Cell Biology & Immunology, University of North Texas Health Science Center, Fort Worth, Texas, United States of America; 3 Department of Neurosurgery, Beijing Tiantan Hospital, Beijing Neurosurgical Institute, Capital Medical University, Beijing, China; Albany Medical College, UNITED STATES

## Abstract

Astrocytes outnumber neurons and serve many metabolic and trophic functions in the mammalian brain. Preserving astrocytes is critical for normal brain function as well as for protecting the brain against various insults. Our previous studies have indicated that methylene blue (MB) functions as an alternative electron carrier and enhances brain metabolism. In addition, MB has been shown to be protective against neurodegeneration and brain injury. In the current study, we investigated the protective role of MB in astrocytes. Cell viability assays showed that MB treatment significantly protected primary astrocytes from oxygen-glucose deprivation (OGD) & reoxygenation induced cell death. We also studied the effect of MB on cellular oxygen and glucose metabolism in primary astrocytes following OGD-reoxygenation injury. MB treatment significantly increased cellular oxygen consumption, glucose uptake and ATP production in primary astrocytes. In conclusion our study demonstrated that MB protects astrocytes against OGD-reoxygenation injury by improving astrocyte cellular respiration.

## Introduction

The brain is almost exclusively dependent on an uninterrupted supply of glucose and oxygen in order to generate energy to meet its physiological requirements. Due to this high energy demand and lack of energy reserves in the brain, short disturbances in the availability of oxygen and glucose can quickly impair energy production and cause metabolic failure [1]. Impaired cerebral metabolism is one of the major features of various neurological disorders including Alzheimer’s disease [2], neuroglycopenia [3] and especially ischemic stroke [1, 4]. During ischemic stroke, interruption of cerebral blood flow dramatically reduces the availability of oxygen and glucose to brain cells. Consequently, within minutes of glucose and oxygen depletion cellular ATP production is severely hampered. This compromises bioenergetic metabolism [5] and energy-dependent processes, such as ionic homeostasis and reuptake of excitatory amino acids, resulting in excitotoxicity, cytotoxic edema and, ultimately, cell death [6]. With thrombolytic therapy, reperfusion of the ischemic brain can be achieved in eligible stroke patients. However, in some circumstances, reperfusion does not reestablish physiological energy metabolism but further deteriorates the injured brain [7, 8] by increasing reactive oxygen species (ROS) production [9] and secondary waves of excitotoxicity [1, 10]. Therefore, a vascular approach to restoring energy substrates as a therapeutic intervention is greatly limited for the treatment of ischemic stroke. A cellular approach to maintenance of bioenergetic metabolism might provide additional treatment for ischemic stroke patients.

Methylene Blue (MB) was synthesized in 1876 and has been used to treat methemoglobinemia, cyanide poisoning and malaria [11]. MB has been shown to attenuate mitochondrial dysfunction under stress [12], increase mitochondrial complex IV activity [13, 14], and delay senescence of human fibroblasts *in vitro* [13]. The unique redox nature of MB enables it to shuttle electrons directly from mitochondrial complex I to Cytochrome C, bypassing complex I/III blockage, thereby preventing electron leakage and superoxide production, and improving mitochondrial respiration [14–16]. Indeed, MB has been shown to improve cerebral blood flow, increase glucose uptake and the cerebral metabolic rate of oxygen consumption under normoxia and hypoxia *in vivo* [16]. MB has been recently studied in various CNS disorders and reported to be protective in rodent models of ischemic stroke [17], Parkinson’s disease [22], Alzheimer’s disease [18, 19], and traumatic brain injury [20].

Astrocytes are the most abundant cells in mammalian brain vastly outnumbering neurons [21]. They possess a unique cyto-anatomical and phenotypic attributes that optimally positions them around neurons and vessels. Astrocytes also play a key role in neurovascular interaction coupling neuronal activity and cerebral blood flow (CBF), which facilitates an uninterrupted supply of oxygen and glucose to meet the high metabolic demands of neurons [22, 23]. In addition, there is increasing evidence indicating that astrocytes are active players in brain energy production, utilization, and storage via astrocyte-neuron metabolic coupling [24, 25]. Astrocytes have been shown to support neuronal metabolism by producing lactate through glycolysis and activation of glycogen catabolism [26]. This neurovascular and metabolic coupling appears to play an important role in the control of neuronal activity in the brain. Impairments in astrocyte metabolism are increasingly being recognized as important contributors to neuronal dysfunction and neurodegenerative processes [27–29]. The goal of the current study was to test the hypothesis that MB protects astrocytes against oxygen glucose deprivation & reoxygenation induced cell death by improving cellular respiration.

## Materials and Methods

### Primary astrocyte cultures

All procedures performed during preparation of primary astrocyte cultures from C576bL6 mice were approved by the Institutional Animal Care and Use Committee of the University of North Texas Health Science Center (UNTHSC) Fort Worth. Primary astrocyte enriched cultures were prepared from 1 day old C57BL6 pups as previously described [30]. Briefly, day old mouse pups were anesthetized by hypothermia followed by decapitation with sharp surgical scissor. Under aseptic conditions the cerebral cortices were dissected and meninges were removed. Cortical tissue was then digested in 0.25% trypsin (Sigma) at 37°C for 20 min. A single cell suspension was prepared by repeated pippeting of the digested cortical tissue through 3 different bore sized Pasteur Pipette. The cell suspension was strained through 40 μM size cell strainer and plated in a 10 cm culture plate in high glucose Dulbecco’s Modified Eagle’s Medium, (DMEM with 4500 mg/l Glucose, 4 mM l-glutamine, 1 mM sodium pyruvate, Thermo Scientific) containing 10% Fetal Bovine serum and Streptomycin (10,000 μg/ml)-Penicillin (10,000 units/ml) and cultured in a humidified incubator at 37°C a with 5% CO_2_ for two weeks. Once the cultures plates became 90% confluent, the plates were constantly shaken for 48hr in a CO_2_ incubator at 37°C to eliminate microglia and other types of cell contaminants.

### *In Vitro* oxygen glucose deprivation model

**Fig 1:**
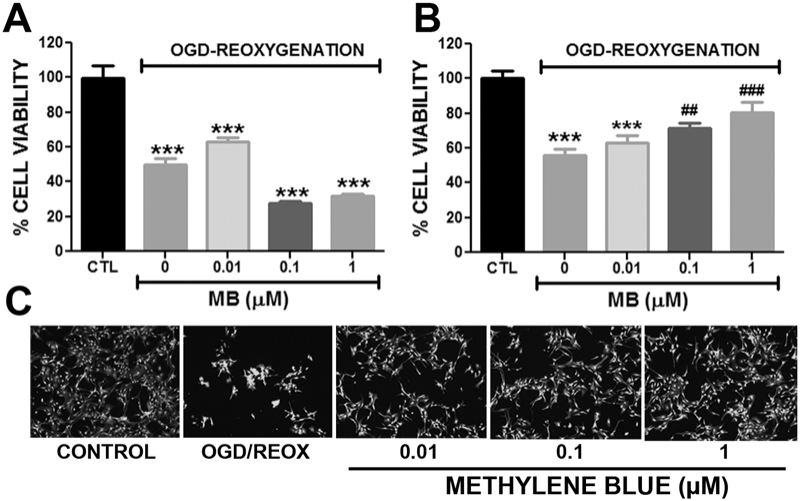
Methylene blue protects astrocytes against OGD-reoxygenation induced cell death. Quantitative analysis of astrocyte viability by Calcein AM assay **A.** Exposure of primary astrocytes to OGD-reoxygenation condition caused significant loss in viability and MB administration during OGD increased cell death. **B.** Administration of MB (0.1 μM and 1 μM) during reoxygenation significantly protected astrocytes from OGD-reoxygenation induced cell death. **C.** Representative images of Calcein AM staining at 24 hours after reoxygenation with or without MB treatment. *** p < 0.0001 Vs. CTL. **#** p < 0.05, **##** and **###** p < 0.0001 Vs. OGD-reoxygenation control /0 μM MB.

Astrocytes were cultured in DMEM (11 mM Glucose, 1 mM pyruvate, 4 mM L-glutamine, Thermo Scientific) for 3 days before experiment. For OGD, the cells were washed twice with sterile Phosphate Buffer Saline (PBS) and incubated in glucose, pyruvate and FBS free DMEM (Thermo Scientific) for 6hr in an anaerobic chamber with 0.1% O_2_ and 5% CO_2_ at 37°C. During reoxygenation, astrocyte media was supplemented with D-Glucose (11 mM), pyruvate (1 mM) and with different concentrations of MB and incubated in 5% CO_2_, 95% air at 37°C for 24hr. At the end of the experiment the viability of astrocytes was determined by Calcein AM assay using a Tecan infinite M200 plate reader and images were taken with Zeiss Observer Z1 microscope (Fig 1).

### Oxygen consumption rate

**Fig 2:**
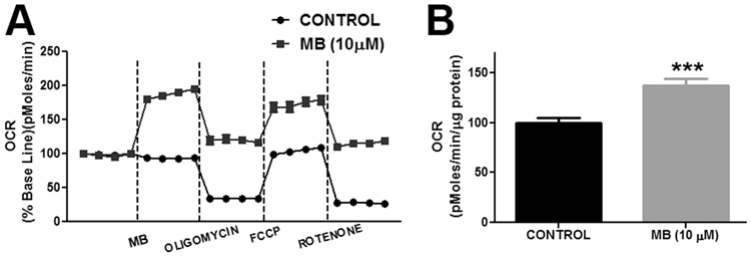
Methylene blue increases oxygen consumption rate (OCR) in primary astrocytes. **A.** Depictions are the changes in OCR in astrocytes following vehicle or MB (10 μM) treatment. MB increased OCR in astrocytes at all-time points. **B.** Quantitative analysis of OCR in control and MB (10 μM) treated astrocytes before oligomycin treatment. MB (10 μM) significantly increased OCR in astrocytes compared to control. *** p < 0.0001 Vs. Control.

Oxygen consumption rate was determined as previously described [31]. Briefly, primary astrocytes were seeded in a XF24 plate at a density of 60,000 cells/well and cultured for 2 days to form an even monolayer. On the day of the experiment the culture media was replaced with XF24 media and incubated for 1hr in a non CO_2_ incubator at 37°C. In the accompanying cartridge of XF24 plate provided in the assay 100X concentration of Rotenone (final concentration 100 nM), FCCP (final concentration 300 nM), Oligomycin (final concentration 1 μg/ml), and MB (final concentration 0.1, 1 and 10 μM), were loaded. The XF24 plate and cartridge were loaded into Seahorse Bioscience XF24 Extracellular Flux Analyzer and drugs were injected at specified time points and oxygen consumption rate was determined (Fig 2). Protein concentration of each sample was determined using Pierce 660 nm Protein assay reagent (Thermo Scientific) and was used to normalize respective oxygen concentration rates.

### Fluorescence-lifetime imaging microscopy (FLTIM)

**Fig 3:**
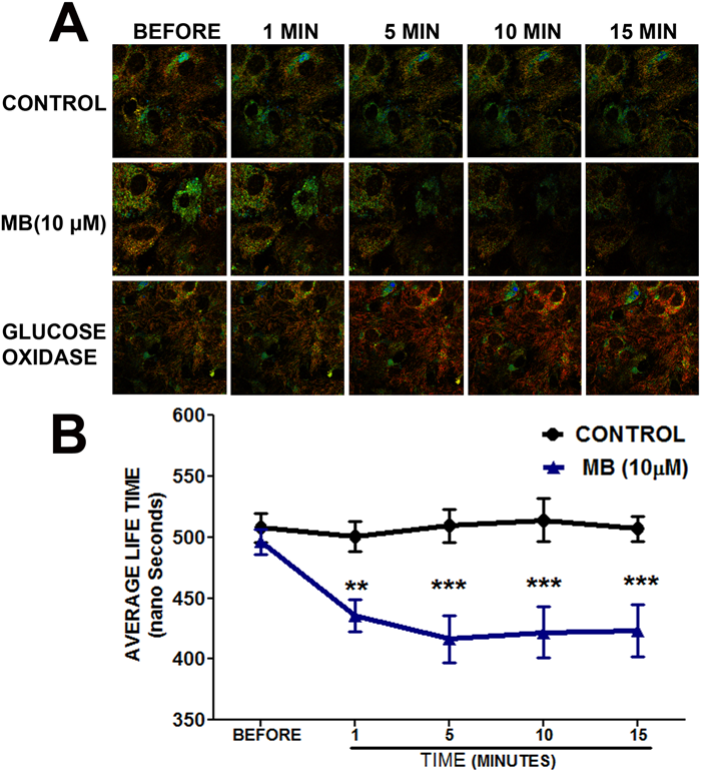
Methylene blue increases intracellular oxygen flux in astrocytes. **A.** Representative image of Ruthenium (II) fluorescence lifetime obtained using FLTIM. All the groups had similar fluorescence before treatment. Ruthenium (II) fluorescence lifetime was shorter in MB (10 μM) treated astrocytes as compared with control. As a positive control, Glucose oxidase (GO) increased Ruthenium (II) fluorescence lifetime in astrocytes. **B.** Quantitative analysis demonstrated that MB treatment significantly decreased Ruthenium (II) fluorescence lifetime in astrocytes. **, *** p < 0.0001 Vs. control.

Astrocytes were cultured on a 35 mm cell culture dish with a cover glass in DMEM (11 mM Glucose, 1 mM pyruvate, 4 mM L-glutamine, Thermo Scientific) with 10% FBS for 2 days. For FLTIM the astrocytes were incubated in Tris (2, 2′-bipyridyl) dichlororuthenium (II) hexahydrate (120 μM in PBS, Sigma) for 2hr. The cells were then washed twice and incubated in sterile Dulbecco’s Phosphate Buffered Saline (DPBS) with 10 mM Dextrose (Sigma). MB and of glucose oxidase (GO, 5 units/mL) were diluted in DPBS from respective stock solutions and added to the dishes during microscopy. Time resolved images were obtained on a confocal MicroTime 200 (Picoquant GmbH, Berlin) system (Fig 3). Excitation was provided from a 470 nm pulsed diode laser (PicoQuant) operating at a 320 kHz repetition rate, and it was reflected off of a 490 nm dichroic plate into an Olympus IX71 inverted microscope. The light passed through an Olympus 60X 1.2 NA objective, and the collected fluorescence was filtered by a 488 nm long wave pass, interference filter before passing through a 50 μm confocal pinhole. The light path terminated at a hybrid photomultiplier assembly detector, also from PicoQuant. The time response of this detector, including the width of the laser pulse, was estimated to be less than 120 ps. The signal from the detector was routed into a PicoHarp 300 (PicoQuant) time correlated single photon counting (TCSPC) module which time-tagged each photon with a resolution of 512 ps per bin. Fluorescence decay curves were analyzed using a single component, exponential, tail-fitting routine which was performed by the software SymPhoTime, v. 5.3.2 from PicoQuant (Fig 3).

### Superoxide analysis

**Fig 4:**
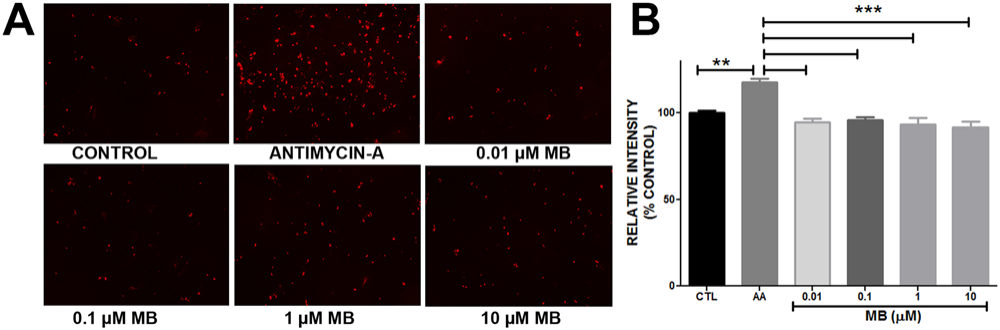
Methylene blue does not increase superoxide production. **A.** Representative image depicting MitoSOX fluorescence in Control, Antimycin-A (AA) and different concentrations of Methylene blue treatment. **B**. Quantitative analysis of MitoSOX assay results. Antimycin-A (50 μM) significantly increased superoxide production in astrocytes compared to control and MB treatment. MB did not increase superoxide production in astrocytes. **, *** p < 0.0001.

Mitochondrial superoxide production in the astrocytes was determined according to manufacturer’s instruction using MitoSOX (Molecular Probes). Astrocytes cultured for 24hrs were washed once with sterile PBS and treated with either MB or Antimycin-A (50 μM) for 20 min. At the end of 20 min astrocytes were washed once with PBS and incubated with MitoSOX (5μM) for 10 min at 37°C. MitoSOX fluorescence was quantified using a Tecan infinite M200 plate reader (Excitation/emission maxima: ~510/580 nm) and images were taken with Zeiss Observer Z1 microscope (Fig 4).

### Glucose uptake

**Fig 5:**
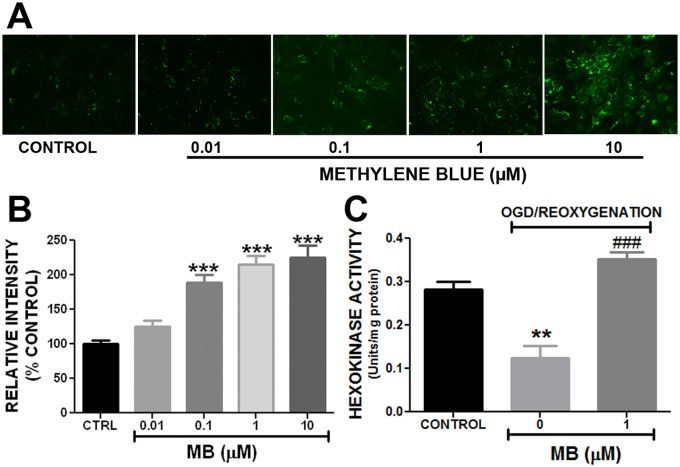
Methylene blue increases glucose uptake and hexokinase activity in astrocytes. **A.** Representative image of 2-NBDG fluorescence in control and MB treated astrocytes under normoxic condition. **B.** Quantitative analysis of 2-NBDG uptake demonstrated that MB concentration-dependently increased 2-NBDG uptake in astrocytes under normoxic condition. **C.** Quantitative analysis of hexokinase activity in astrocytes. Methylene blue (1 μM) significantly restored OGD induced loss of hexokinase activity. **, *** p < 0.0001 Vs. Control. **###** p < 0.0001 Vs. OGD-reoxygenation control / 0 μM MB.

Glucose uptake in astrocytes was determined using glucose analog 2-NBDG as previously described [16]. Briefly, astrocytes were cultured in 96 well culture plates (5000 cells/well) and 25 mm cover slip (30,000 cells /coverslip) in DMEM (11 mM Glucose, 1 mM pyruvate, 4 mM L-glutamine, Thermo Scientific). On the day of experiment the cells were washed twice and incubated in glucose-free Krebs Ringer HEPES (KRH) buffer (129 mM NaCl, 5 mM NaHCO_3_, 4.8 mM KCl, 1.2 mM KH_2_PO_4_, 1 mM CaCl_2_, 1.2 mM MgCl_2_, 10 mM HEPES; pH 7.4) for 30 min. The astrocytes were then incubated in glucose free KRH buffer containing 100 μM of 2-NBDG and different concentrations of MB for 5 min. The astrocytes were washed thrice with KRH buffer and glucose uptake in astrocytes in 96 well plates was determined using a Tecan Infinite M200 plate reader (Excitation/Emission for 2-NBDG ~465/540 nm). The coverslips were used for microscopy using Zeiss Observer Z1 microscope (Fig 5).

### Hexokinase activity

Activity of hexokinase (HK) was measured at 37°C in a Flexstation (Molecular devices) as described earlier with minor modification [32]. The assay principle involved enzymatic conversion of glucose to glucose 6-phosphate by hexokinase which is further converted to gluconate 6-phosphate by glucose-6-phosphate dehydrogenase during which one micromole of NAD^+^ is reduced per one unit activity/min. The change in absorbance is then measured using a spectrophotometer. For the assay, 0.67 M Glucose, 16.5 mM Adenosine 5'Triphosphate, 6.8 mM NAD and 300 IU/ml of glucose-6-phosphate dehydrogenase were prepared in 0.05 M Tris*HCl buffer, pH 8.0 with 13.3 mM MgCl2. In a 96 well plate, to each well 228 μl of Tris MgCl2 buffer, 50 μl of glucose and 10 μl each of ATP, NAD and glucose-6-phosphate dehydrogenase were added. Finally 10 μl of cell lysate was added and the change in absorbance was determined at 340 nm. ΔA/min was then calculated from initial linear portion of the curve (Fig 5C). Protein concentration of the samples was determined by colorimetric analysis using Pierce 660 nm Protein assay reagent (Thermo Scientific) and Tecan Infinite M200 plate reader. Enzyme activities were expressed as units per milligram of protein (Fig 5).

### ATP assay

**Fig 6:**
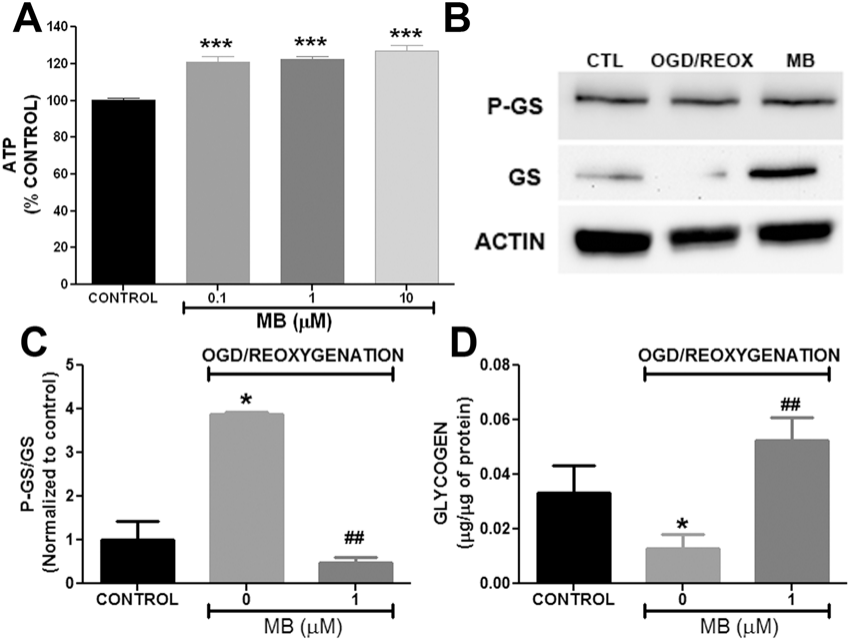
Methylene blue increases ATP production and attenuate phosphorylation of glycogen synthase in astrocytes. **A.** Quantitative analysis of ATP production in MB treated astrocytes at 60 min after reoxygenation. MB significantly increased ATP production in primary astrocytes after transient OGD. **B**. Representative Western blots of glycogen synthase (GS), Phospho glycogen synthase (PGS) and actin in astrocytes 24 hours after OGD-reoxygenation. **C**. Quantitative analysis of Western blots demonstrated that ratio P-GS/GS was significantly increased in astrocytes following OGD-reoxygenation which was significantly reduced in MB (1 μM) treated astrocytes. **D**. Quantitative analysis of glycogen content in astrocytes. At 24 hours following OGD-reoxygenation, astrocytes had less glycogen content compared to normoxia control. MB (1 μM) treated astrocytes had significantly higher glycogen content compared to non-MB treated cells following OGD-reoxygenation. * p < 0.05; *** p < 0.0001 Vs. Control. ## p < 0.001 Vs. OGD-reoxygenation control / 0 μM MB.

The change in cellular ATP levels was determined using ATP kit (Invitrogen) as previously described [31]. Briefly, the astrocytes were cultured for 24hr in a 6 well culture plate at a density 2.5 X 10^4^ cells/well in DMEM (11 mM Glucose, 1 mM pyruvate, 4 mM L-glutamine, Thermo Scientific) at 37°C. The cells were washed twice with glucose and pyruvate free KRH buffer and incubated in KRH buffer for 30 min. The KRH buffer was replaced with culture medium (DMEM) with different concentrations of MB and incubated for either 30 or 60 min. The cells were then detached with trypsin (Sigma) and washed twice with PBS and lysed with 100 μl of ATP assay buffer (500 mM Tricine buffer, pH 7.8, 100 mM MgSO_4_, 2 mM EDTA, and 2 mM sodium azide, 1% Triton X-100). ATP reaction buffer (100 μl, 30 μg/ml D-luciferin, 20 μM DTT, and 25 μg/ml luciferase) was added to 10 μl of cell lysate and the luminescence was measured using a Tecan Infinite M200 plate reader. The ATP values were determined from a standard curve and normalized to the protein content of each sample (Fig 6).

### Western blot analysis

At the end of OGD-reoxygenation the cells were lysed in a cell lysis buffer (20 mM of Tris, 100 mM NaCl, 1 mM DiSodium-EDTA, 0.5% Triton-X) with protease and phosphatase inhibitors (1:100). Protein content of the samples was determined using Pierce 660 nm Protein assay reagent (Thermo Scientific). The samples were resolved on 10% SDS gel and transferred to a nitrocellulose membrane. The membranes were incubated overnight in primary antibodies against glycogen synthase (1:500; rabbit, Cell Signaling), Phospho-glycogen synthase (1:500; rabbit, Cell Signaling), and actin (1:1000; mouse, Santa Cruz) followed by secondary antibody (Jackson Immunoresearch, West Grove, PA). Using Biospectrum 500 UVP imaging system chemiluminescence signal was detected (Fig 6). The protein densities of glycogen synthase, Phospho-glycogen synthase were normalized to actin density.

### Glycogen Analysis

Total glycogen in astrocytes was determined by using Abcam Glycogen assay kit according to the manufacturer’s instruction. For sample preparation following OGD-reoxygenation with or without MB, the astrocytes were detached with 0.25% trypsin (Sigma), washed twice with PBS and resuspended in 150 μl of double distilled water. The cells were lysed by sonication and boiled for 5min at 95°C. In a 96 well plate, 20 μl of sample was incubated in glycogen hydrolysis buffer containing glucoamylase enzyme provided with the kit for 30 min at room temperature. After incubation, OxiRed Probe, glycogen hydrolysis enzyme mix, glycogen development enzyme mix were added to samples and incubated for another 30 min. The glycogen content was determined by using fluorometric assay (excitation/emission = 535/587 nm) and the fluorescence was measured using Tecan Infinite M200 plate reader. Corrections for background glucose were made in all the samples and the corrected fluorescent readings were applied to standard curve and amount of glycogen in each well was determined (Fig 6). The glycogen content of each sample was normalized to their respective protein content and plotted using Graph Pad Prism 5.

### Statistical analysis

Statistical analysis was performed using Graph Pad Prism 5. Results are expressed as mean ± standard error of mean (SEM). When comparing two groups a t-test was used to identify any significant differences. For comparison of multiple groups, two-way analysis of variance was used and post-hoc Bonferroni analysis was done to identify the significant differences. p < 0.05 was considered as statistically significant.

## Results

### MB protects astrocytes against transient OGD-reoxygenation induced cell death

*In vitro* 6hr OGD and 24hr reoxygenation caused a significant cell death (50%) in primary astrocytes and MB treatment during OGD did not confer any protection, rather increased cell death induced by OGD (Fig 1). However, when administered immediately after reoxygenation, MB conferred a significant (p<0.05%) protection against transient OGD induced cell death (Fig 1).

### MB increases oxygen consumption rate in astrocytes

We determined oxygen consumption rate (OCR) in astrocytes by sequential injection of MB/ vehicle, oligomycin, FCCP and rotenone. Our results showed that MB and vehicle treated astrocytes had significantly different OCR at all-time points of the experiment. MB (10 μM) administration induced a significant increase in OCR in astrocytes compared to vehicle treated cells (Fig 2). The results obtained using the Seahorse Bioscience XF24 Extracellular Flux analyzer indicated the changes in oxygen concentration in the media. We further determined the effect of MB treatment on intracellular oxygen using an oxygen sensing dye, Tris (2, 2′-bipyridyl) dichlororuthenium (II) hexahydrate and FLTIM. Ruthenium’s (II) fluorescence is quenched in the presence of high oxygen concentration [33]. Therefore, the lifetime of ruthenium fluorescence provides an indirect measurement of intracellular oxygen concentration. FLTIM indicated that the average fluorescence lifetime of ruthenium was significantly reduced following MB treatment as compared to vehicle control (Fig 3), indicating that MB treatment significantly increased oxygen flux into astrocytes in agreement with the increased OCR as observed in Seahorse Flux analyzer experiment. The sensitivity of Ruthenium (II) to cellular oxygen status was confirmed by glucose oxidase (GO), which consumes intracellular oxygen, hence, creates a low intracellular oxygen tension and increases average lifetime of Ruthenium (II)’s fluorescence. During reoxygenation, perturbation of mitochondrial electron transfer chain (ETC) results in overproduction of superoxide [34]. We further investigated the effect of MB on superoxide production using Mitosox assay. We found that MB treatment did not increase superoxide production, while, as predicted, antimycin-A significantly increased astrocytes superoxide production (Fig 4).

### Methylene Blue increases glucose uptake and restore hexokinase activity after OGD in astrocytes

To determine if MB increases glucose uptake in astrocytes we used 2-NBDG, a fluorescent glucose analogue, which cells take up via glucose transporters. Once taken up, 2-NBDG is metabolized very slowly. This enables quantification by measuring its fluorescence intensity. Fluorescent microscopy and quantitative analysis of 2-NBDG uptake revealed that MB increased glucose uptake in astrocytes in a concentration dependent manner (Fig 5). MB significantly increased glucose uptake in astrocytes by 89%, 200% and 225% at the concentrations of 0.1 μM, 1 μM and 10 μM, respectively.

Hexokinase is the rate limiting enzyme in glycolysis which phosphorylates glucose and traps it for cellular utilization. We observed the effect of MB on hexokinase activity in astrocytes following OGD insult. Our results showed that following transient OGD there was a significant reduction of hexokinase activity in astrocytes which was significantly abolished by MB treatment (Fig 5).

### MB promotes ATP production and glycogen synthesis in primary astrocytes

We determined ATP production in astrocytes following MB treatment. MB significantly increased ATP production in astrocytes following 60 min treatment in normoxic condition (Fig 6). Glycogen is a high molecular-weight glucose polymer which is used as energy storage in the brain. We observed the effect of MB on glycogenesis in astrocytes. Glycogen synthase (GS) is the enzyme involved in the conversion of glucose to glycogen. This reaction is inhibited by phosphorylation of GS. Our Western blot analysis indicated that transient OGD followed by reoxygenation inhibited glycogenesis by decreasing GS which was attenuated by MB treatment (Fig 6). This result was further confirmed by analysis of total glycogen in astrocytes. Results from glycogen analysis showed that glycogen content in astrocytes following OGD-reoxygenation was significantly less compared to normoxia control and that MB treatment significantly increased glycogen content in astrocytes after OGD (Fig 6).

## Discussion

In the current study we determined the protective role of MB against energy deprivation in primary astrocytes and delineated its underlying mechanism. Using an in *vitro* model of transient OGD, which resembles ischemia reperfusion injury, we demonstrated that MB is protective in astrocytes against energy deprivation-induced cell death when administered immediately after restoration of glucose and oxygen.

Ischemic stroke causes a reduction of cerebral blood flow, resulting in ischemic brain damage. Prompt restoration of blood flow is crucial to reduce or prevent neurological damage in ischemic stroke patients. Paradoxically, reperfusion can exacerbate brain damage through reperfusion injury [35]. Most notably, electrons leaking from the electron transport chain (ETC), mostly at complex I and complex III, react with oxygen to yield superoxide anion, which can be converted into other ROS [36, 37]. With the primary ETC complexes damaged by ischemia, restoration of oxygen after reperfusion leads to a surge in ROS production which in turn facilitates a vicious cycle of accelerated mitochondrial damage, excitotoxicity, lipid peroxidation, and inflammation [38]. Oxidative stress is the essential mechanism for ischemia/reperfusion and has been proposed as the key culprit for ischemic stroke damage [39]. However, the failure of the SAINT-II trial has raised concern regarding the free radical scavenger approach for the treatment of ischemic stroke [39, 40]. Our current study indicates that MB differs from the traditional antioxidants in that its protective action was only afforded when administered after reoxygenation. When administered during glucose and oxygen depletion, MB in fact exaggerates the damage. The identified unique protective action of MB against OGD-reoxygenation damage in astrocytes is in line with our previous studies that MB functions as an alternative mitochondrial electron carrier and enhances mitochondrial oxidative phosphorylation [14, 15].

Our current study demonstrates that MB significantly increases oxygen influx and oxygen consumption rate in astrocytes, indicating that MB enhances astrocyte glucose catabolism and mitochondrial oxidative phosphorylation. Glucose is the principal and obligatory fuel of the mammalian brain required for high energy metabolism. In the event of glucose oxygen deprivation as seen during ischemia, subsequent loss of ATP can quickly lead the brain tissue to irreversible metabolic failure. In the present study we demonstrated that MB significantly increased glucose uptake in astrocytes in a concentration dependent manner. Importantly, the increase in glucose uptake induced by MB is accompanied by an increase in hexokinase activity. Hexokinase is the first enzyme in glucose metabolism and is responsible for irreversible conversion of glucose to glucose-6-phosphate. The increase in hexokinase activity induced by MB indicates that MB indeed enhances glucose metabolism. In astrocytes an increase in ATP production was consistently observed upon MB treatment. Besides its crucial function in the glycolytic pathway, hexokinase also plays an important role in the cellular survival mechanism by interacting with mitochondrial BCl-2, an antagonist of cell death (BAD) and modulates apoptosis in response to glucose deprivation. Hexokinase isoforms especially HK II is known to elicit anti-apoptotic function and protect astrocytes and neurons from hypoxia. The present study demonstrates a significant reduction in hexokinase activity following OGD in astrocytes which could lead to reduction in glucose utilization. MB treatment prevents the reduction and maintains the hexokinase activity which improves glucose metabolism following OGD-reoxygenation.

The greatly impaired cerebral ATP production during ischemia is not completely restored even after tissue reperfusion and reduction in oxidative metabolism of glucose continues to persists in later stage of reperfusion [41, 42]. In our study we observed that MB increases astrocyte glucose uptake, hexokinase function, and oxygen consumption as well as ATP production. These effects are particularly beneficial in the post ischemic brain which primarily depends on glycolysis for ATP production and in turn causes acidosis which is detrimental to the surviving brain cells [43, 44]. We speculate that the bioenergetic action of MB is a direct result of its alternate mitochondrial electron carrier action, which bypasses the mitochondrial complex I-III blockage thus improving mitochondrial respiration [15]. This notion is further supported by the detrimental action of MB against ischemia reperfusion injury when administered during OGD. During ischemia, acceleration of oxygen consumption and glucose metabolism might further exaggerate ischemic damage. However, the deleterious effect of MB on ischemia when administered during OGD should not be interpreted as a contradiction for using MB before thrombolytic therapy in ischemic stroke patients. MB might be limited in its ability to reach the ischemic territory without thrombolysis induced reperfusion. Indeed, a recent study demonstrated that MB treatment delays progression of perfusion-diffusion mismatch to infarct in a permanent ischemic stroke model [45].

In the current study, we observed that ATP production was improved in astrocytes in response to MB treatment. However, the increase in ATP upon MB treatment was not in proportion to the increase in glucose uptake and hexokinase activity. Mitochondrial complex I and III serve as the major proton pumps which generate a proton gradient across the mitochondrial inner membrane, the ultimate driving force for mitochondrial ATP production. We speculate that, as an alternative mitochondrial electron carrier bypassing complex I-III, MB might have a partial mitochondrial uncoupling effect which renders a disproportional increase of ATP production in relation to the increase of glucose uptake and hexokinase activity after MB treatment. In addition, the disproportional increase of ATP production might be due to the increase of glycogenesis upon MB treatment. Glycogen, a polymer of glucose, is exclusively stored in adult brain astrocytes [46] and it has been reported that almost 50% of brain glucose is taken up by astrocytes under physiological conditions [47]. Studies have indicated that astrocyte glycogen stores can preserve neuronal function and viability in the conditions of restricted ATP availability [24, 25, 48]. We found a significant decrease of glycogenesis in astrocytes following glucose and oxygen deprivation evidenced by reduction of both active unphosphorylated glycogen synthase and glycogen content in astrocytes which was attenuated by MB treatment. Therefore, the enhancement of glycogenesis and glycogen stores might also contribute the disproportional increase of ATP production and glucose uptake upon MB treatment. As mentioned above, ischemia induced injury at cellular level is reflected as severe derailment of cellular metabolic processes. This impaired process is not restored even after reperfusion as a result the cells undergo apoptosis leading to growth of infarct during chronic stages of injury. Our study indicated that administration of MB early during reoxygenation alleviated the bioenergetic deficiency and we presume this caused the cells to reestablish metabolic integrity which in long term prevented metabolic failure induced cell death.

In summary, we demonstrated that MB exerts a unique protective effect in astrocytes against transient oxygen glucose deprivation-reoxygenation induced cell death. MB provides protection when administered during reoxygenation, while it exaggerates OGD-reoxygenation damage when administered during OGD. In addition, MB enhances astrocyte glucose metabolism and mitochondrial oxidative phosphorylation, increasing astrocyte energy storage. The unique protective action of MB in astrocytes might provide a novel cellular approach to maintaining brain bioenergetic metabolism as a combined therapy with thrombolysis for the treatment of ischemic stroke.

## Supporting Information

S1 Dataset Data for Fig 1. Methylene blue protects astrocytes against OGD-reoxygenation induced cell death.
(XLS)

S2 Dataset Data for Fig 2. Methylene blue increases oxygen consumption rate (OCR) in primary astrocytes.
(XLS)

S3 Dataset Data for Fig 3. Methylene blue increases intracellular oxygen flux in astrocytes.
(XLS)

S4 Dataset Data for Fig 4. Methylene blue does not increase superoxide production.
(XLS)

S5 Dataset Data for Fig 5. Methylene blue increases glucose uptake and hexokinase activity in astrocytes.
(XLS)

S6 Dataset Data for Fig 6. Methylene blue increases ATP production and attenuate phosphorylation of glycogen synthase in astrocytes.
(XLS)
